# The effect of various breath‐hold techniques on the cardiorespiratory response to facial immersion in humans

**DOI:** 10.1113/EP090531

**Published:** 2022-11-30

**Authors:** Matthew J. Burley, Jamie Blackwell, Bert Bond, Craig Williams, Francis B. Stephens

**Affiliations:** ^1^ Department of Sport and Health Sciences College of Life and Environmental Sciences University of Exeter Exeter UK

**Keywords:** apnoea, breath‐hold, cardiovascular, end‐tidal, haemoglobin, respiratory

## Abstract

Repeated maximal breath‐holds have been demonstrated to induce bradycardia, increase haematocrit and haemoglobin and prolong subsequent breath‐hold duration by 20%. Freedivers use non‐maximal breath‐hold techniques (BHTs) to improve breath‐hold duration. The aim of this study was to investigate the cardiorespiratory and haematological responses to various BHTs. Ten healthy men (34.5 ± 1.9 years) attended five randomized experimental trials and performed a 40 min period of quiet rest or one of three BHTs followed by a maximal breath‐hold challenge during facial immersion in water at 30 or 10°C. Cardiovascular and respiratory parameters were measured continuously using finger plethysmography and breath‐by‐breath gas analysis, respectively, and venous blood samples were collected throughout. Facial immersion in cold water caused marked bradycardia (74.1 vs. 50.2 beats/min after 40 s) but did not increase breath‐hold duration compared with warm water control conditions. Facial immersion breath‐hold duration was 30.8–43.3% greater than the control duration when preceded by BHTs that involved repeated breath‐holds of constant duration (*P* = 0.021), increasing duration (*P* < 0.001) or increasing frequency (*P* < 0.001), with no difference observed between BHTs. The increased duration of apnoea across all three BHT protocols was associated with a 6.8% increase in end‐tidal O_2_ and a 13.1% decrease in end‐tidal CO_2_ immediately before facial immersion. There were no differences in blood pressure, cardiac output, heart rate, haematocrit or haemoglobin between each BHT and control conditions (*P* > 0.05). In conclusion, the duration of apnoea can be extended by manipulating blood gases through repeated prior breath‐holds, but changes in cardiac output and red blood cell mass do not appear essential.

## Abstract (highlights)

- **What is the central question of this study?**
  What is the effect of three repeated breath‐hold techniques routinely used by freedivers, thought to manipulate arterial partial pressures of O_2_ and CO_2_, on the cardiorespiratory and haematological response to breath‐holding during facial immersion?
- **What is the main finding and its importance?**
  All three techniques increased breath‐hold by a similar duration, probably owing to the similar marked increase in end‐tidal O_2_ and decrease in end‐tidal CO_2_ observed in all three trials before facial immersion. These were the only cardiorespiratory changes that were consistently manipulated before the maximal breath‐hold. This would suggest that pronounced bradycardia and vasoconstriction of selective vascular beds are probably not obligatory for prolonging breath‐hold duration.

## 1 INTRODUCTION

Three to five repeated maximal breath‐holds have been demonstrated to induce bradycardia, increase haematocrit (Hct) and haemoglobin (Hb) and increase the subsequent breath‐hold time by ≤20% (Baković et al., 2003; M. Richardson et al., 2005). These physiological responses are consistent with the mammalian dive reflex (MDR) on facial immersion in cold water (Foster & Sheel, 2005; Nepal et al., 2014; Schagatay et al., 2001). Peripheral vasoconstriction is also characteristic of the dive reflex and, together with bradycardia, is exacerbated with a reduction in water temperature during facial immersion, probably via stimulation of the trigeminal nerve (Nepal et al., 2014; Paulev et al., 1990). However, there is limited evidence to show that a lower water temperature prolongs breath‐hold time in humans (Foster & Sheel, 2005), which would suggest that pronounced bradycardia and vasoconstriction of selective vascular beds are perhaps not obligatory for prolonging breath‐hold duration. Erythrocyte release from the spleen is considered to be the cause of increased Hct and Hb, because splenic contraction and a 14% reduction in the splenic volume are observed during breath‐holding in humans and are more pronounced in trained ‘freedivers’ (deep water diving without the use of breathing apparatus; Baković et al., 2003). Indeed, studies in splenectomized individuals demonstrate a reduced breath‐hold time in some (Baković et al., 2003), but not all cases (Schagatay et al., 2001), regardless of the dive reflex. Thus, it is still unclear whether these physiological responses to repeated maximal breath‐holds contribute to prolonged breath‐hold duration.

It is common practice for human freedivers to use repeated breath‐hold techniques (BHTs) before a dive to manipulate blood gases and prolong subsequent dive duration. It is well established that breath‐hold time is almost doubled by breath‐holding with hyperoxic gas mixtures to increase the partial pressure of arterial oxygen ($P_{aO_{2}}$) (Ferris et al., 1946; Gross et al., 1976) or by preceding breath‐holding by voluntary or mechanical hyperventilation to lower the partial pressure of arterial carbon dioxide ($P_{\mathrm{aC}O_{2}}$) (Klocke & Rahn, 1959). However, to our knowledge, whether repeated breath‐holds manipulate $P_{aO_{2}}$ and $P_{\mathrm{aC}O_{2}}$ and contribute to the prolongation of subsequent breath‐hold duration has not been investigated. Thus, the aim of the study was to investigate the effect of three different repeated BHTs routinely used by freedivers, which are thought to manipulate $P_{aO_{2}}$ and $P_{\mathrm{aC}O_{2}}$ to varying degrees, on the cardiorespiratory and haematological responses to breath‐holding during facial immersion. By comparing these responses observed during facial immersion in warm and cold water, it would also be possible to provide insight into the contribution, if any, of the MDR to breath‐hold prolongation. We hypothesized that a BHT that uses repeated and progressive near‐maximal breath‐holds would create the largest cardiorespiratory perturbation and induce the most prolonged breath‐hold duration.

## 2 METHODS

### 2.1 Ethical approval

This study was approved by the Research Ethics Committee of the Department of Sport and Health Sciences (reference 180906/A/01), University of Exeter, in accordance with the latest version of the *Declaration of Helsinki* (2013), except for registration in a database. All subjects were verbally informed about the nature and risks of the experiment in conjunction with a participant information sheet and consent form before written informed consent was obtained.

### 2.2 Participants

Ten healthy males (34.5 ± 1.9 years of age, 179.5 ± 1.2 cm in height and weighing 85.5 ± 1.7 kg) participated in this study. All participants were non‐smokers and were not suffering from any cardiovascular, neurological, respiratory or metabolic diseases. Furthermore, none of the participants had been splenectomized, and they were not taking any prescribed medication. All participants were asked to report to the human physiology laboratories at the University of Exeter on five occasions in a randomized order, each separated by ≥1 week, having abstained from strenuous physical activity and alcohol for the previous 24 h and from food for the previous 2 h (Ghiani et al., 2016; Schagatay & Lodin‐Sundström, 2014).

### 2.3 Protocol

On arrival in the laboratory, participants’ height and weight were measured. Participants were seated in a comfortable upright position (Cicolini et al., 2011) in a temperature‐controlled laboratory (21°C). A finger plethysmograph (human non‐invasive blood pressure monitor, NIBP Nano Interface; ADInstruments, Colorado Springs, CO, USA) was fitted via two appropriately sized finger cuffs on the left hand for continuous measurement of beat‐to‐beat pulse pressure and heart rate (HR) and indirect calculation of mean arterial blood pressure (MABP), stroke volume (SV), cardiac output ($\dot{Q}$) and total peripheral resistance (TPR) throughout the experiment. A height‐correction unit (NIBP Nano; ADInstruments) was placed in line with the bottom of the participant's heart for blood pressure autocalibration when adjusting the position for facial immersion. A cannula was inserted into an antecubital forearm vein of the right arm for repeated blood sampling. To prevent clotting, the cannula was routinely flushed with saline. Lastly, a face mask (Korr; Medical Technologies) connected to a spirometer (ADInstruments) was placed on the subject to measure end‐tidal (ET) O_2_ and ETCO_2_ as a proxy for $P_{aO_{2}}$ and $P_{\mathrm{aC}O_{2}}$, respectively (McSwain et al., 2010). Breath gases were sampled at the mouthpiece and analysed by a calibrated gas analyser (ML206; ADInstruments). Labchart (v.7; ADInstruments) was used to calculate breath‐by‐breath tidal volumes, ETO_2_, ETCO_2_ and all ET cardiovascular measurements.

**FIGURE 1:**
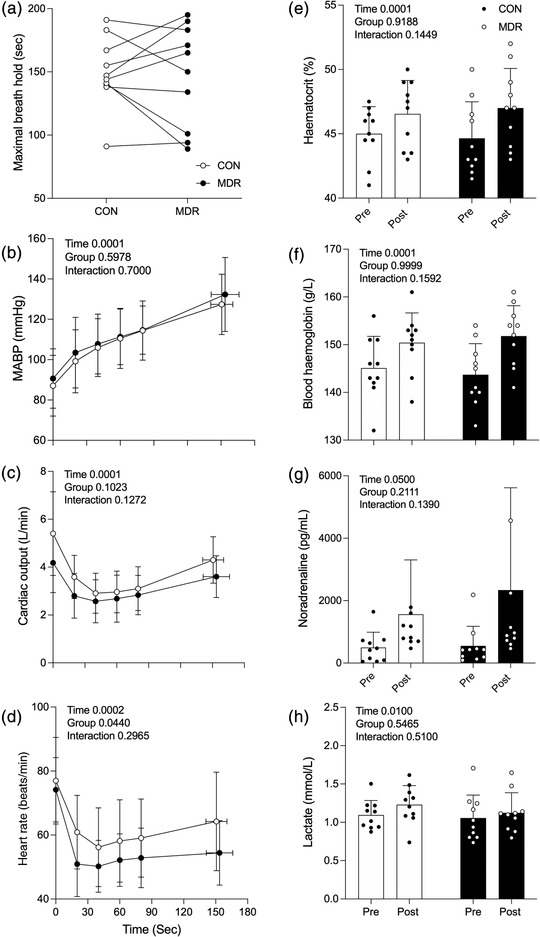
Maximal breath‐hold duration (a) and mean arterial blood pressure (MABP; b), cardiac output (c), heart rate (d), haematocrit (e), blood haemoglobin (f), plasma noradrenaline (g) and plasma lactate (h) responses to a maximal breath‐hold during facial immersion in warm water (30°C; CON) or cold cold (10°C; MDR) conditions. Blood and plasma measurements were made before (Pre) and immediately after (Post) facial immersion. Values are the mean ± SD for *n* = 10. Main effects of repeated‐measures ANOVA are presented in each panel

After 20 min of quiet rest, participants were asked conduct one of five 40 min protocols consisting of either quiet rest for the two control protocols (CON and MDR) or one of three BHTs in a randomized order, which were designed by a member of the research team (M.J.B.) in line with similar protocols used by freedivers and aimed at producing divergent $P_{aO_{2}}$ and $P_{\mathrm{aC}O_{2}}$ (Figure 1). Repeated breath‐holds (RBH) maintained consistent 1 min breath‐hold durations separated by decreasing rest intervals (by 10 s from 2:50 min) over the 40 min period (total breath‐holds 15, total breath‐hold duration 15 min) in order to raise $P_{aO_{2}}$ and lower $P_{\mathrm{aC}O_{2}}$ gradually. Prolonged breath‐holds (PBH) required increasing breath‐hold duration (increasing by 10 s every breath‐hold from 30 s) separated by consistent 1 min rest duration (total breath‐holds 15, total breath‐hold duration 25 min) in order to maintain the number of breath‐holds but increase breath‐hold duration. Increasing breath‐holds (IBH), which required increasing both the number and duration of breath‐holds (total breath‐holds 23, total breath‐hold duration 20:30 min), beginning at 20 s and adding 10 s to the breath‐hold duration after every three breath‐holds until reaching 1:20 min, followed by two final breath‐holds of 1:30 min, separated by increasing recovery periods (nine sets at 40 s, three sets at 50 s, 11 sets at 60 s). Ten seconds before each breath‐hold, the participant was instructed to perform a ‘breath‐up’ consisting of a deep inhalation (∼80% of self‐predicted maximal inspiratory volume), a complete exhalation and a final full inhalation before holding.

After the BHT period was complete, the face mask was removed, and participants were asked to rest for 2 min for recovery of spleen size (Espersen et al., 2002). A nose clip was fitted, followed by the ‘breath‐up’ manoeuvre 10 s before facial immersion (Andersson et al., 2000) in warm water (30°C), during which participants were asked to hold their breath for as long as comfortably possible. To exacerbate the MDR, one of the control protocols (MDR) required facial immersion in cold water (10°C) (Fagius & Sundlof, 1986; Tipton, 2003). Participants responded to a shoulder tap with a hand signal as a safety control measure. At the breakpoint, the face mask was replaced once the face had been dried, and participants rested quietly for a further 60 min.

### 2.4 Sample collection and analysis

Heart rate (in beats per minute), MABP (in milllimetres of mercury), SV (in millilitres per beat), $\dot{Q}$ (in litres per minute), TPR (in milllimetres of mercury) and pre‐breath‐hold ET CO_2_ (ETCO_2_; in milllimetres of mercury) and O_2_ (ETO_2_; in milllimetres of mercury) measurements were collected continuously, with the exception of ETCO_2_ and ETO_2_ during facial immersion, owing to face mask removal. A total of 2 ml of venous blood was obtained every 20 min from the beginning of the protocol and immediately after facial immersion until completion. Blood samples were analysed immediately for Hb concentration (in grams per litre) using a microcuvette system (HemoCue Hb 201+; HemoCue, Sweden) and Hct (as a percentage) using a 75 mm microfuge tube (SciQuip), with centrifugation for 2 min at 4,000*g* and measurement with a micro‐haematocrit reader (Hawksley). The remainding whole blood was transferred to a vacutainer containing 10.2 mg of potassium EDTA centrifuged for 8 min at 3,250*g*, and plasma was stored at −80°C for future analysis. Noradrenaline was analysed by an enzyme‐linked immunosorbent assay (ELISA) method using a commercially available kit (noradrenaline ELISA; Abnova, Taipei City, Taiwan). Absorbance was measured and quantified against known standards using an EnSpire 2300 plate reader (Perkin Elmer, MA, USA). Plasma lactate was analysed using a 2500 glucose/lactate analyser (YSI).

### 2.5 Data and statistical calculations

Cardiorespiratory data were reported every minute during CON and MDR or at time points that matched ET gas measurements immediately before the ‘breath‐up’ manoeuvre (Pre‐BH) and at the stipulated breakpoint of each breath‐hold (Post‐BH) during the three BHT protocols.

Maximal breath‐hold duration was analysed using a one‐way ANOVA. All other data were measured using a two‐way ANOVA with the factors of condition and time. The assumptions to conduct these parametric statistics were confirmed before running all statistics. When a significant main effect was observed for interaction, a post‐hoc Sidak test was used with Geisser–Greenhouse correction for sphericity where necessary. Data were analysed using commercially available software (Prism v.8; GraphPad Software, San Diego, CA, USA). All variables are presented as the mean ± SD. A *P*‐value of <0.05 was considered significant.

## 3 RESULTS

### 3.1 Comparisons between control and MDR during facial immersion

Baseline measures MABP (87.1 vs. 90.6 mmHg; Figure 1b), $\dot{Q}$ (5.4 vs. 4.2 L/min; Figure 1c), HR (77.0 vs. 74.1 beats/min; Figure 1d), Hct (45 vs. 44.6%; Figure 1e), Hb (145.1 vs. 143.7 g/L; Figure 1f), noradrenaline (498.2 vs. 551.1 pg/ml; Figure 1g) and lactate (1.1 mmol/L; 1.1 mmol/L; Figure 1h) were similar between CON and MDR, respectively. Change over time occurred for all measures (MABP *P* < 0.001, $\dot{Q}$ *P* < 0.001, HR *P* < 0.001, Hct *P* < 0.001, Hb *P* < 0.001, noradrenaline *P* = 0.050 and lactate *P* = 0.010; Figure 1b–h); however, no significant changes were observed when comparing CON with MDR (Figure 1).

### 3.2 Maximal breath‐hold duration during facial immersion

**FIGURE 2:**
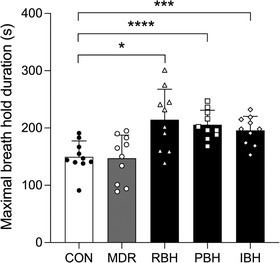
Maximal breath‐hold duration during facial immersion in warm water (30°C) after 40 min of quiet rest (CON) or a 40 min repeated breath‐hold technique in which each breath‐hold duration was kept constant (RBH) or gradually prolonged (PBH) or the number of breath‐holds was increased (IBH). Values are the mean ± SD for *n* = 10. ^*^ *P* < 0.05, ^***^ *P* < 0.01 and ^****^ *P* < 0.001 significantly greater than CON

The mean maximal breath‐hold duration for participants was 149.7 ± 27.8, 147.2 ± 40.6, 214.5 ± 53.3, 205.7 ± 25.2 and 195.8 ± 24.6 s for CON, MDR, RBH, PBH and IBH, respectively (RBH *P* = 0.021, PBH *P* < 0.001 and IBH *P* < 0.001, all vs. CON; Figure 2). All the participants increased breath‐hold duration from CON to PBH and IBH compared with eight participants in RBH. No difference in maximal breath‐hold duration was observed between MDR and CON (Figures 1a and 2).

### 3.3 Respiratory response

**FIGURE 3:**
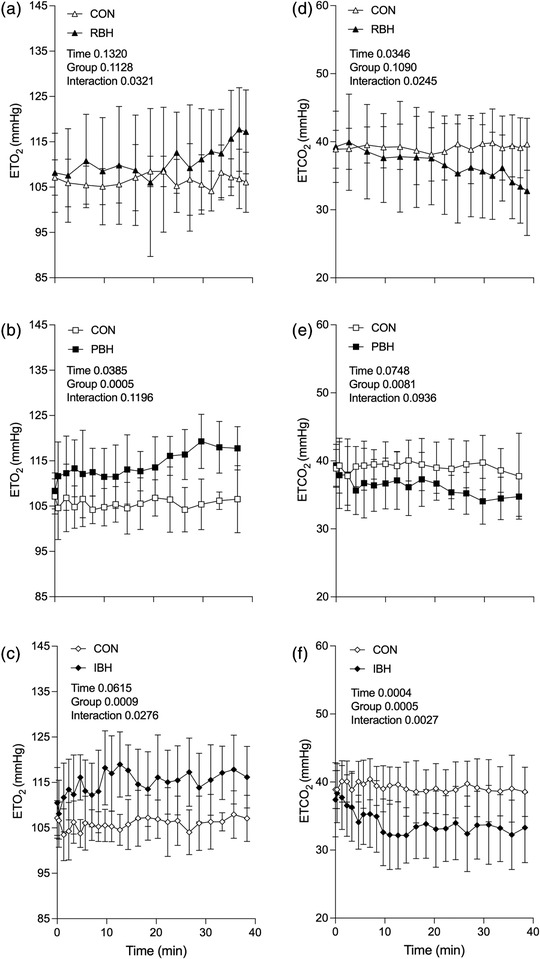
End‐tidal (ET) O_2_ (a–c) and CO_2_ (d–f) during a 40 min repeated breath‐hold technique in which each breath‐hold duration was kept constant (RBH; a,d) or gradually prolonged (PBH; b,e) or the number of breath‐holds was increased (IBH; c,f). Each breath‐hold technique measurement is compared with a corresponding value during 40 min of quiet rest (CON). Values are the mean ± SD for *n* = 10. Main effects of repeated‐measures ANOVA are presented in each panel

Baseline ETO_2_ was similar between participants during all protocols (CON 107.1 ± 3.9 mmHg, MDR 108.7 ± 7.1 mmHg, RBH 108.2 ± 8.7 mmHg, PBH 108.4 ± 4.0 mmHg and IBH 110.5 ± 7.8 mmHg; Figure 3a–c). During Pre‐BH, overall ETO_2_ increased (RBH *P* = 0.032 and IBH *P* = 0.028, Figure 3a,c), with further increases observed immediately before facial immersion (RBH 117.2 ± 9.3 mmHg, *P* = 0.025; PBH 117.2 ± 9.3 mmHg, *P* = 0.013; and IBH 116.2 ± 6.8 mmHg, n.s.; Figure 3a–c), and no differences were observed during MDR when compared with CON. Post‐BH ETO_2_ results revealed a decrease during PBH and IBH compared with CON (*P* < 0.001) with further differences observed immediately before facial immersion (RBH 100.5 ± 9.0 mmHg,*P* = 0.030; and PBH 60.9 ± 21.5 mmHg, *P* = 0.003). At the 48 min point, ETO_2_ had returned to baseline in all protocols, and no differences were observed during the recovery period.

Baseline ETCO_2_ was similar between participants during all protocols (CON 38.9 ± 2.8 mmHg, MDR 37.6 ± 1.6 mmHg, RBH 39.3 ± 5.2 mmHg, PBH 39.3 ± 3.1 mmHg and IBH 37.4 ± 5.5 mmHg; Figure 3d–f). During Pre‐BH, overall ETCO_2_ decreased (RBH *P* = 0.024 and IBH *P* = 0.003), with further increases observed immediately before facial immersion (RBH 32.8 ± 6.5 mmHg, *P* = 0.016; and IBH 33.3 ± 5.1 mmHg, n.s.; Figure 3d,f) compared with CON. No differences were observed when comparing PBH (34.7 ± 2.9 mmHg, n.s.; Figure 3e) or MDR with CON. Conversely, during Post‐BH, only PBH increased overall ETCO_2_ compared with CON (*P* < 0.001), and no differences were observed immediately before facial immersion in all protocols. At the 48 min time point, PBH ETCO_2_ was acutely lower than CON (34.6 ± 4.1 mmHg, *P* = 0.006), and no difference was observed 1 min later and thereafter. All other protocols had returned to baseline by the 48 min time point, and no differences were observed during the remaining recovery period.

### 3.4 Cardiovascular responses

**FIGURE 4:**
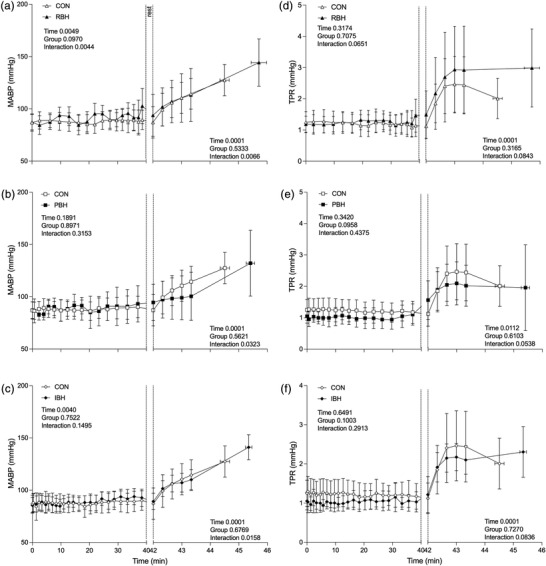
Mean arterial blood pressure (MABP; a–c) and total peripheral resistance (TPR; d–f) during a 40 min repeated breath‐hold technique in which each breath‐hold duration was kept constant (RBH; a,d) or gradually prolonged (PBH; b,e) or the number of breath‐holds was increased (IBH; c,f). Each breath‐hold technique measurement is compared with a corresponding value during 40 min of quiet rest (CON). After a 2 min ‘rest’ period, the response to a maximal breath‐hold during facial immersion in warm water (30°C) is presented on the right‐hand *x*‐axis. Values are the mean ± SD for *n* = 10. Main effects of repeated‐measures ANOVA are presented in each panel

Baseline MABP was similar between participants during all protocols (CON 86.9 ± 8.3 mmHg, MDR 84.6 ± 6.0 mmHg, RBH 87.3 ± 7.4 mmHg, PBH 87.1 ± 8.0 mmHg and IBH 85.4 ± 7.4 mmHg; Figure 4a–c). During Pre‐BH MABP, only RBH was higher than CON (*P* = 0.004; Figure 4a) both overall and immediately before facial immersion (RBH 102.7 ± 16.9 mmHg, *P* = 0.0476), whilst no differences were observed in PBH (93.2 ± 17.4 mmHg; Figure 4b), IBH (92.8 ± 8.3 mmHg; Figure 4c) or MDR. Post‐BH BHT overall results were all higher than CON (RBH *P* = 0.023, PBH *P* < 0.001 and IBH *P* < 0.001; Figure 4a–c); however, no differences were observed immediately before facial immersion or MDR when compared with CON. During facial immersion, all BHTs were significantly different from CON (RBH *P* = 0.007, PBH *P* = 0.032 and IBH; *P* = 0.016; Figure 4a–c), whilst no difference was observed in MDR (Figure 1b). All BHTs returned to their baseline at the 48 min time point, with no changes throughout the recovery period and no differences compared with CON.

Baseline TPR was similar between protocols (CON 1.2 ± 0.3 mmHg, MDR 1.1 ± 0.2 mmHg, RBH 1.2 ± 0.3 mmHg, PBH 1.0 ± 0.2 mmHg and IBH 1.0 ± 0.2 mmHg; Figure 4d–f). During Pre‐BH, no differences were observed between BHTs and MDR compared with CON (Figure 4d–f). Post‐BH revealed differences across all three BHTs compared with CON (RBH *P* < 0.001, PBH *P* = 0.0002 and IBH *P* < 0.001); however, no differences were observed immediately before facial immersion (RBH 1.5 ± 0.5 mmHg, PBH 1.1 ± 0.3 mmHg and IBH 1.0 ± 0.3 mmHg) or during MDR compared with CON. During facial immersion, all protocols increased TPR (CON 2.4 ± 0.9 mmHg, MDR 2.7 ± 1.4 mmHg, RBH 2.9 ± 1.4 mmHg, PBH 2.0 ± 0.7 mmHg and IBH 2.1 ± 0.4 mmHg, *P* < 0.001; Figure 4d–f), but no difference was observed compared with CON. All protocols had returned to baseline at the 48 min time point, and no differences was observed throughout the recovery period.

**FIGURE 5:**
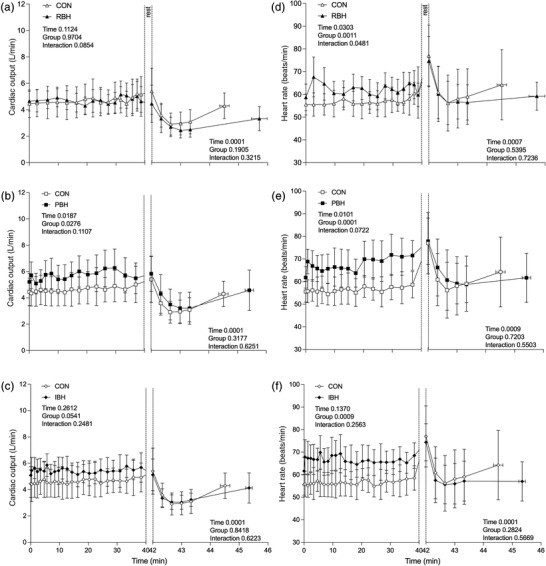
Cardiac output (a–c) and heart rate (HR; d–f) during a 40 min repeated breath‐hold technique in which each breath‐hold duration was kept constant (RBH; a,d) or gradually prolonged (PBH; b,e) or the number of breath‐holds was increased (IBH; c,f). Each breath‐hold technique measurement is compared with a corresponding value during 40 min of quiet rest (CON). After a 2 min ‘rest’ period, the response to a maximal breath‐hold during facial immersion in warm water (30°C) is presented on the right‐hand *x*‐axis. Values are the mean ± SD for *n* = 10. Main effects of repeated‐measures ANOVA are presented in each panel

Baseline $\dot{Q}$ was not different between protocols (CON 4.5 ± 1.0 L/min, MDR 5.0 ± 0.8 L/min, RBH 4.7 ± 0.7 L/min, PBH 5.2 ± 0.6 L/min and IBH 5.1 ± 0.7 L/min; Figure 5a–c). During Pre‐BH, BHTs (RBH 4.6 ± 1.1 L/min, PBH 5.5 ± 1.2 L/min and IBH 5.7 ± 1.1 L/min; Figure 5a–c) and MDR displayed no differences compared with CON. During Post‐BH, all BHTs were different compared with CON (RBH *P* < 0.001, PBH *P* < 0.001 and IBH *P* = 0.009), whilst CON and MDR elicited no change. Although change over time occurred in all protocols, no differences were observed before facial immersion during BHTs (Figure 5a–c) or MDR (Figure 1c) compared with CON. This shows no contribution of $\dot{Q}$ difference towards breath‐hold duration. During facial immersion, all protocols displayed decreases (CON 3.1 ± 0.9 L/min, MDR 2.8 ± 0.8 L/min, RBH 2.5 ± 0.6 L/min, PBH; 3.2 ± 1.2 L/min and IBH 3.2 ± 0.5 L/min, *P* < 0.001; Figure 5a–c), with no differences observed compared with CON. All protocols returned to baseline, and no differences were observed at the 48 min time point or during the recovery period.

Baseline HR was similar between participants during all protocols (CON 55.6 ± 5.1 beats/min, MDR 57.1 ± 7.0 beats/min, RBH 58.7 ± 5.9 beats/min, PBH 61.8 ± 4.7 beats/min and IBH 61.6 ± 6.0 beats/min; Figure 5d–f). During Pre‐BH, RBH overall HR was different (*P* = 0.048; Figure 5d), and differences were observed immediately before facial immersion (PBH 71.5 ± 6.6 beats/min, *P* = 0.005; Figure 5b), whilst no differences was observed during IBH (68.5 ± 5.7 beats/min; Figure 5f) or MDR when compared with CON. During Post‐BH, overall HR in RBH demonstrated a difference compared with CON (*P* = 0.032), whilst no differences were observed in all other protocols immediately before facial immersion. During facial immersion, HR decreased in all protocols, but only MDR was lower than CON (*P* = 0.038; Figure 1d) at the 40 s time point, demonstrating that cold water intensifies bradycardia without necessarily increasing breath‐hold duration. All BHTs returned to their baseline at the 48 min time point, with no changes throughout the recovery period and no differences compared with CON.

### 3.5 Haematological parameters

**FIGURE 6:**
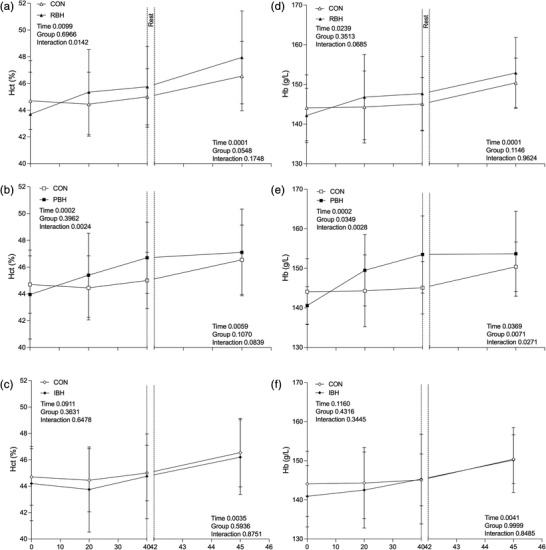
Haematocrit (Hct; a–c) and blood haemoglobin concentration (Hb; d–f) during a 40 min repeated breath‐hold technique in which each breath‐hold duration was kept constant (RBH; a,d) or gradually prolonged (PBH; b,e) or the number of breath‐holds was increased (IBH; c,f). Each breath‐hold technique measurement is compared with a corresponding value during 40 min of quiet rest (CON). After a 2 min ‘rest’ period, the response to a maximal breath‐hold during facial immersion in warm water (30°C) is presented on the right‐hand *x*‐axis. Values are the mean ± SD for *n* = 10. Main effects of repeated‐measures ANOVA are presented in each panel

Baseline Hb and Hct were similar between participants during all protocols (CON 144.1 ± 8.3 g/L and 44.7 ± 2.1%, MDR 142.4 ± 7.3 g/L and 44 ± 2.3%, RBH 142.2 ± 6.9 g/L and 43.7 ± 4.0%, PBH 140.6 ± 4.8 g/L and 43.9 ± 3.3% and IBH 140.9 ± 7.8 g/L and 44.2 ± 2.8%; Figure 6). During the BHT phase, overall Hb and Hct displayed an increase during RBH (Hct *P* = 0.014; Figure 6a) and PBH (Hct *P* = 0.002 and Hb *P* = 0.003; Figure 6b,e), with further increases observed during PBH (RBH 147.7 ± 9.4 g/L, *P* = 0.491 and 45.7 ± 3.0%, *P* = 0.459; and PBH 153.5 ± 9.8 g/L, *P* = 0.003 and 46.7 ± 2.7%, *P* = 0.100; Figure 6e) compared with CON, whilst no differences were observed during IBH (145.3 ± 11.5 g/L, *P* = 1.000 and 44.7 ± 3.2%, *P* = 0.977; Figure 6c,f) or MDR before facial immersion. An increase occurred across all protocols during facial immersion (CON 150.4 ± 6.3 g/L and 46.5 ± 2.6%; MDR 151.8 ± 6.4 g/L and 47.0 ± 3.1%; RBH 152.9 ± 8.9 g/L and 47.9 ± 3.5%; PBH 153.7 ± 10.7 g/L and 47.1 ± 3.2%; and IBH 150.2 ± 8.3 g/L and 46.2 ± 2.8%), and a difference was observed during PBH (*P* = 0.027; Figure 6e) compared with CON. Observing the overall recovery period, RBH (Hb *P* = 0.038 and Hct *P* = 0.035) and PBH (Hb *P* = 0.019) displayed differences from CON. No differences were observed across all protocols at the 65 min time point compared with CON; however, IBH (Hb *P* = 0.031) was different from CON at the 85 min time point.

### 3.6 Noradrenaline response

**FIGURE 7:**
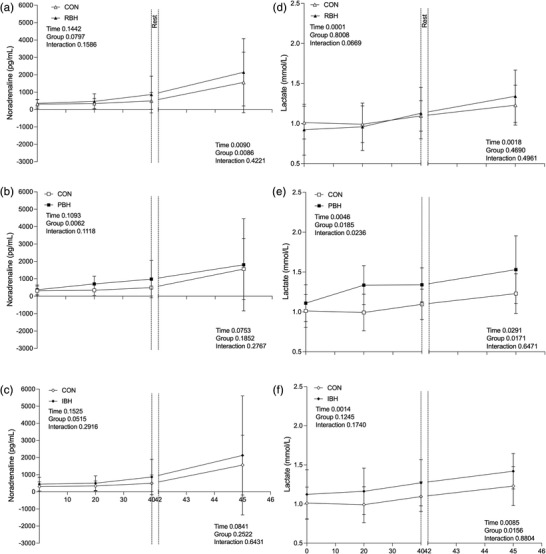
Plasma noradrenaline (a–c) and lactate (d–f) concentrations during a 40 min repeated breath‐hold technique in which each breath‐hold duration was kept constant (RBH; a,d) or gradually prolonged (PBH; b,e) or the number of breath‐holds was increased (IBH; c,f). Each breath‐hold technique measurement is compared with a corresponding value during 40 min of quiet rest (CON). After a 2 min ‘rest’ period, the response to a maximal breath‐hold during facial immersion in warm water (30°C) is presented on the right‐hand *x*‐axis. Values are the mean ± SD for *n* = 10. Main effects of repeated‐measures ANOVA are presented in each panel

Baseline noradrenaline was similar during all protocols (CON 312.8 ± 268.0 pmol/L, MDR 307.9 ± 179.9 pmol/L, RBH 360.0 ± 210.3 pmol/L, PBH 374.0 ± 271.4 pmol/L and IBH 449.7 ± 399.2 pmol/L; Figure 7a–c). During the BHT phase, overall noradrenaline did not increase in all BHTs or MDR, and no differences were observed before facial immersion (MDR 551.1 ± 625.7 pmol/L; Figure 1g; RBH 865.6 ± 1059.8 pmol/L, PBH 982.6 ± 1071.7 pmol/L and IBH 864.8 ± 1026.5 pmol/L; Figure 7a–c) compared with CON (498.2 ± 488.6 pmol/L; Figure 7a–c). A difference was observed at the 20 min time point during PBH (705.2 ± 439.0 pmol/L, *P* < 0.001; Figure 7b) compared with CON (344.1 ± 294.4 pmol/L). During facial immersion, overall increases in noradrenaline were observed in all protocols; however, only RBH displayed any difference from CON at the 45 min time point (2,146.3 ± 1,934.7 pmol/L, *P* = 0.026; Figure 7a) despite the remaining protocols displaying higher mean values (MDR 2,338.3 ± 3,280.2 pmol/L, PBH 1,806.1 ± 2,648.3 pmol/L and IBH 2,126.9 ± 3,476.2 pmol/L; Figure 7b,c). Observing overall recovery, RBH (*P* = 0.026) was different from CON. In all protocols, noradrenaline had returned to baseline at the 65 min point, and no other differences were observed.

### 3.7 Lactate response

Baseline lactate was similar between participants during all protocols (CON 1.0 ± 0.2 mmol/L, MDR 0.9 ± 0.2 mmol/L, RBH 0.9 ± 0.3 mmol/L, PBH 1.1 ± 0.2 mmol/L and IBH; 1.1 ± 0.3 mmol/L; Figure 7d–f). During the BHT phase, overall lactate increased during PBH (*P* = 0.024; Figure 7e), and a difference was observed at the 20 min time point (1.3 ± 0.2 mmol/L, *P* = 0.020; Figure 7e) compared with CON. No differences were observed immediately before facial immersion in all protocols (MDR 1.1 ± 0.3 mmol/L; Figure 1h; RBH 1.3 ± 0.3 mmol/L, PBH 1.3 ± 0.2 mmol/L, IBH and 1.3 ± 0.3 mmol/L; Figure 7d–f) compared with CON (1.1 ± 0.2 mmol/L). During facial immersion, increases were observed in all protocols (CON 1.2 ± 0.2 mmol/L, MDR 1.1 ± 0.3 mmol/L; Figure 1h; RBH 1.3 ± 0.3 mmol/L, PBH 1.5 ± 0.4 mmol/L and IBH; 1.4 ± 0.2 mmol/L; Figure 7d–f), but no differences were observed compared with CON. During overall recovery, no differences were observed compared with CON. Only PBH showed any differences during recovery at the 65 (*P* = 0.042) and 85 min time points (*P* = 0.027). No other differences compared with CON or changes compared with baseline were observed.

## 4 DISCUSSION

The aim of the present study was to investigate the effect of three different repeated BHTs routinely used by freedivers, thought to manipulate $P_{aO_{2}}$ and $P_{\mathrm{aC}O_{2}}$ to varying degrees, on the cardiorespiratory and haematological responses to breath‐holding during facial immersion in warm water. We hypothesized that a BHT that uses repeated near‐maximal breath‐holds would create the largest cardiorespiratory perturbation and induce the longest breath‐hold duration. However, contrary to this hypothesis, all BHTs increased breath‐hold duration to a similar degree. These observations were likely attributable to the similar increase in ETO_2_ and decrease in ETCO_2_ observed in all three trials before facial immersion compared with the control conditions. Indeed, these were the only cardiorespiratory changes that were consistently manipulated before the maximal breath‐hold. Thus, previous reports that an increase in circulating Hct and Hb and a reduction in HR are required for breath‐hold prolongation with prior breath‐holding, where blood gases have been manipulated, perhaps require further investigation. For example, HR was elevated before performing a maximal breath‐hold after the three breath‐hold test trials and, although facial immersion in cold water induced a marked bradycardia commonly associated with the MDR, the breath‐hold duration was not greater than for facial immersion in warm water.

Performing a maximal breath‐hold during facial immersion in water at 30°C in the present study caused an increase in MABP and TPR, a decrease in $\dot{Q}$, SV and HR and an increase in circulating Hct, Hb, noradrenaline and lactate. These findings are in line with other reports (Foster & Sheel, 2005; Parkes, 2006; Schagatay, 2014) and with the theory that the increase in MABP is attributable to a sympathetically mediated peripheral vasoconstriction, whereas the decline in $\dot{Q}$ could be a subsequent baroreceptor response or independently chemoreceptor mediated (Paulev et al., 1990; Perini et al., 1998). Consistent with what would be expected from stimulating the MDR, facial immersion in cold water at 10°C caused a further reduction in HR. However, this marked bradycardia did not prolong maximal breath‐hold duration and would perhaps indicate that the dive reflex is advantageous only when accompanied by other physiological circumstances that occur during deep diving, such as hyperbaria. Indeed, HR and $\dot{Q}$ were either similar or higher than control values before and during facial immersion for each of the BHTs, which is in agreement with other studies (Heusser et al., 2009). Thus, other cardiorespiratory or haematological responses observed during a maximal breath‐hold might confer a milieu favourable to prolonging subsequent breath‐hold duration.

The BHTs used in the present study resulted in a 30.8–43.3% prolongation of breath‐hold duration during a subsequent breath‐hold test. To our knowledge, this is the largest increase in breath‐hold duration recorded in the literature after prior voluntary breath‐holding and the first documented use of these techniques in a laboratory experiment that has measured cardiorespiratory and haematological responses. Baković et al. (2003) and M. Richardson et al. (2005) demonstrated that a 20% increase in breath‐hold duration after three to five repeated maximal breath‐holds was associated with a 1.4% increase in Hb concentration, probably attributable to a 14% reduction in splenic volume during the prior breath‐holds. The increase in Hb, Hct and splenic contraction, which appears to be more pronounced under hypercapnia (M. X. Richardson et al., 2012), accounts for ∼60% of breath‐hold prolongation (Schagatay et al., 2001), and greater increases in Hb concentration are associated with long‐term breath‐hold training (Lemaitre et al., 2009; Schagatay, 2014; Zoretic et al., 2014). The present study provided a unique scenario to provide further insight into the relationship between Hb concentration and breath‐hold duration. Although comparatively larger increases in Hb were observed before (5.5%) and during (2.1%) the maximal breath‐hold in the PBH compared with CON conditions in the present study, there was no difference in Hb concentration in the IBH conditions compared with CON conditions, despite an increase (30.8–43.3%) in maximal breath‐hold duration during both sets of conditions. Furthermore, the increase in Hct before and during the maximal breath‐hold followed a similar pattern in both PBH and IBH. Thus, although an increase in circulating Hb and Hct was observed during maximal breath‐holds, as found in other studies (Baković et al., 2003; Espersen et al., 2002; M. Richardson et al., 2005), the magnitude of increase before or during apnoea does not appear to influence breath‐hold duration. This observation suggests that other factors could be more important; for example, changes in blood gases.

It is well established that breath‐hold duration is almost doubled by breath‐holding with hyperoxic gas mixtures to increase $P_{aO_{2}}$ (Ferris et al., 1946; Gross et al., 1976) or by preceding breath‐holding by voluntary or mechanical hyperventilation to lower $P_{\mathrm{aC}O_{2}}$ (Klocke & Rahn, 1959). However, whether repeated breath‐holds manipulate $P_{aO_{2}}$ and $P_{\mathrm{aC}O_{2}}$, contributing to prolongation of subsequent breath‐hold duration, has not been investigated. Throughout all three BHTs, there was a marked increase in ETO_2_ and decrease in ETCO_2_ observed before facial immersion and maximal breath‐hold compared with control conditions, which appeared to be the only cardiorespiratory changes consistently manipulated before the maximal breath‐hold. The reason for the consistent changes observed in ETO_2_ and ETCO_2_ after all three techniques is not clear but might be attributable to recovery hyperventilation immediately after every breath‐hold of each technique. Hence, when compared with RBH, the increase in ETO_2_ and decrease in ETCO_2_ occurred earlier during PBH and IBH; both had much shorter recovery periods during the initial breath‐holds and, presumably, less time to normalize blood gases. In line with this theory, the increase in ETO_2_ and decrease in ETCO_2_ in RBH eventually occurred when the recovery periods reduced to a similar duration to those in PBH and IBH (i.e. ∼60 s or less). Given the incremental nature of the increase in ETO_2_ and decrease in ETCO_2_, it would appear, perhaps counterintuitively, that repeated short recovery periods between breath‐holds, in addition to prolonged breath‐hold periods, might have a greater effect on blood gas manipulation and maximal breath‐hold duration. More research on this practice is clearly warranted, but it is interesting to note that PBH, which had longer initial breath‐holds than IBH and short (60 s) recovery periods, appeared to have the earliest changes in circulating Hb, Hct, noradrenaline and lactate, perhaps reflecting greater cardiorespiratory ‘stress’ and recovery hyperventilation.

In conclusion, this is the first study to demonstrate that progressive repeated breath‐holds will manipulate ETO_2_ and ETCO_2_ and, presumably, the associated blood gases and will prolong breath‐hold duration during facial immersion. The increase in maximal breath‐hold duration was similar across all three BHTs and is consistent with that observed anecdotally in freedivers. In contrast, there did not appear to be a clear association with maximal breath‐hold duration and any other cardiovascular or haematological parameters measured across the three techniques, indicating that they do not have a major effect on breath‐hold prolongation. However, it is important to note that the present study was not performed in trained divers, who might have different physiological responses to a maximal breath‐hold after repeated breath‐holds. Indeed, splenic contraction observed during breath‐holding in humans is more pronounced in trained freedivers (Baković et al., 2003). Thus, further research on the physiological effects of these BHTs in trained individuals is clearly required.

## AUTHOR CONTRIBUTIONS

Matthew J. Burley and Francis B. Stephens contributed to the conception and design of the experiment. Matthew J. Burley, Francis B. Stephens and Jamie Blackwell contributed to data collection and analysis. Matthew J. Burley drafted the manuscript. All authors contributed to the interpretation of the data and revision towards important intellectual content. All authors approved the final version of the manuscript, ensuring accuracy and that questions related to the integrity of the research were scrutinized and resolved. All persons designated as authors qualify for authorship, and all those who qualify for authorship are listed.

## CONFLICT OF INTEREST

None declared.

## Supporting information

Statistical Summary Document

Statistical Summary Document

## Data Availability

All data supporting the results of the paper are available as Supporting Information.
